# Narcissoside attenuates atherosclerosis by suppressing CD36-mediated foam cell formation via upregulation of NR4A1

**DOI:** 10.1186/s13020-026-01412-1

**Published:** 2026-05-20

**Authors:** Ziyuan Wang, Lamei Zhou, Xiaorong Zhang, Zhiren Yao, Yaping Huang, Lei Wang, Ke Pan, Jian Zhang, Hao Zhi, Zhiqi Yin

**Affiliations:** 1 School of Traditional Chinese Pharmacy, China Pharmaceutical University, Nanjing, 211198 China; 2 Affiliated Hospital of Integrated Traditional Chinese and Western Medicine, Nanjing University of Chinese Medicine, Nanjing, 210028 China; 3 Department of Rheumatology, Wuxi Hospital of Traditional Chinese Medicine, Wuxi, 214000 China

**Keywords:** Atherosclerosis, Narcissoside, Foam cell, CD36, NR4A1

## Abstract

**Background:**

The formation of foam cells (FCs) is the major contributor to the development of atherosclerosis (AS). *Gynostemma pentaphyllum* is widely used to treat AS and exhibits biological activity against FCs formation. Narcissoside (Nar) is an important component from *G. pentaphyllum* flavonoids. This study aimed to reveal the beneficial effect and underlying mechanism of Nar on inhibiting the formation of FCs in AS.

**Methods:**

The inhibitory effects of Nar on FCs formation were evaluated using Oil Red O staining and flow cytometry in ox-LDL-stimulated THP-1 and BMDMs. High-fat diet (HFD)-induced ApoE^−/−^ mice were administered Nar for 9 weeks to assess its therapeutic efficacy in treating AS. Luciferase reporter gene assays, Western blot, and RT-qPCR were performed to investigate the mechanisms of action of Nar, which were further confirmed through in vitro experiments using siRNA of NR4A1 for reverse validation.

**Results:**

In vitro, Nar attenuated FCs formation as evidenced by reduced intracellular lipid levels and ox-LDL uptake rates. In vivo, Nar effectively improved lipid profiles and serum inflammatory factors. Histological analysis showed that Nar reduced the area of the necrotic core and lipid accumulation in the aortic roots. Mechanistically, Nar inhibited CD36 transcription by enhancing the expression and nuclear translocation of NR4A1. Mutation of the NR4A1 binding site within the CD36 promoter abolished the inhibitory effect of Nar. Additionally, silencing NR4A1 eliminated Nar’s capacity to suppress ox-LDL uptake and downregulate CD36 expression.

**Conclusions:**

Nar attenuated AS by inhibiting CD36-mediated FCs formation through upregulating the expression and nuclear translocation of NR4A1, highlighting it as a promising therapeutic candidate for AS.

**Supplementary Information:**

The online version contains supplementary material available at 10.1186/s13020-026-01412-1.

## Background

Atherosclerosis (AS) is the primary pathological basis of cardiovascular disease, with the characteristic of the accumulation of lipid plaques in the artery walls [1]. The current therapeutic strategy for AS primarily involves the utilization of lipid-lowering medications while AS continues deteriorating even after achieving the target lipid level [2, 3]. Consequently, the development of novel anti-AS drugs is urgently needed in clinical treatment.

Foam cell (FCs) is the crucial part of AS lesions and its formation is the dominant driver of AS [4]. The dysregulation of macrophage cholesterol uptake, mediated by scavenger receptors (SRs), is recognized as the primary cause of FCs formation. Cluster of differentiation 36 (CD36), as a member of the class B scavenger receptor, accounts for approximately 70% of ox-LDL uptake in macrophages [5, 6]. The suppression of CD36 expression significantly attenuated cholesterol uptake and FCs formation [7]. Therefore, targeting CD36 to inhibit FCs formation and suppress lipid uptake represents a promising therapeutic intervention for AS.

The nuclear receptor subfamily 4 group A member 1 (NR4A1) binds with the promoter of target genes through DNA-binding zinc finger motifs and orchestrates heterogeneous biological processes [8]. The knockout of NR4A1 in LDLR^−/−^ mice enhanced macrophage accumulation and accelerated the progression of AS [9]. Furthermore, the overexpression of NR4A1 in macrophages significantly suppressed FCs formation, cholesterol uptake, and CD36 expression [10]. In breast cancer, NR4A1 functioned as a transcription factor to inhibit CD36 expression, thereby attenuating the uptake of fatty acids by cancer cells [11]. Therefore, these investigations suggest that NR4A1 may be crucial in regulating CD36 expression during FCs formation.

As a traditional Chinese medicine, *Gynostemma pentaphyllum* (*G. pentaphyllum*) has been widely used in clinical therapy for hyperlipidemia and AS. Previous studies have elucidated the beneficial role of *G. pentaphyllum* in inhibiting FCs formation which was attributed to Gypenosides (GPs) [12, 13]. Our preliminary experimental findings revealed the beneficial effect of *G. pentaphyllum* flavonoids (GFs) on inhibiting the formation of FCs, which is more effective than GPs. It has been found that several flavonoids derived from GFs have also demonstrated certain efficacy in anti-FCs generation [14]. We screened some flavonoids from GFs on inhibiting FCs generation in vitro and found that Narcissoside (Nar) was the most effective. Furthermore, certain hydroxyl flavonoids have been reported to modulate NR4A1 activity [15]. Therefore, this study aimed to investigate whether Nar attenuates FCs formation and to elucidate the underlying mechanisms.

In this study, we investigated the effect of Nar on inhibiting FCs generation using Oil Red O and flow cytometry in vitro. The therapeutic effect of Nar on AS was evaluated in ApoE^−/−^ mice. The potential molecular mechanisms of Nar were revealed in FCs models using Luciferase reporter gene experiments, immunofluorescence, and siRNA.

## Materials and methods

### Reagents and antibodies

Gypenosides (GPs) and *G. pentaphyllum* flavonoids (GFs) were supplied by Hunan Huabaotong Pharmaceutical Co., Ltd. (Hunan, China). GF-1 (vicenin II), GF-2 (quercetin-3-*O*-β-d-neospheroside), GF-3 (Rutin), GF-4 (Narcissoside), GF-5 (kaempferide 3-*O*-neohesperidoside) were isolated from *Gynostemma pentaphyllum* flavonoids (Fig. S1). Atorvastatin was purchased from Pfizer Pharmaceutical Co., Ltd. (Dalian, China). The ox-LDL (20605ES05) and Dil-ox-LDL (20609ES76) were purchased from Yeasen Biotechnology Co., Ltd (Shanghai, China). RIPA lysis buffer (P0013B), Triton X-100 (P0096), carboxymethylcellulose sodium (CMC-Na, P1373788-01) were from Beyotime (Shanghai, China). Cell counting kit-8 reagents (CCK8, KGA1606), phosphate buffer saline (PBS, KGL2206) and bovine serum albumin (BSA, KGL2314) were obtained from KeyGEN Biotechnology (Nanjing, China). Phorbol 12-myristate 13-acetate (PMA, HY-18739) was purchased from MedChemExpress (New Jersey, USA).

Anti-CD36 (rabbit monoclonal antibody, DF13262), GAPDH (rabbit monoclonal antibody, AF7021) were provided from Affinity Biotechnology (Changzhou, China); Histone H3 (rabbit monoclonal antibody, 17168-1-AP), NR4A1 (rabbit monoclonal antibody, 12235-1-AP), α-SMA (rabbit monoclonal antibody, 14395-1-AP), CD68 (rabbit monoclonal antibody, 30929-1-AP), HRP-conjugated Affinipure Goat Anti-Rabbit IgG (SA00001-2), CoraLite488-conjugated Goat Anti-Rabbit IgG (SA00013-2), CoraLite594-conjugated Goat Anti-Rabbit IgG (SA00013-4) and anti-IgG (30000-0-AP) were purchased from Proteintech (Wuhan, China).

### Cell culture and treatment

Human monocytic leukemia (THP-1) cell was obtained from the China Center for Type Culture Collection (Wuhan, China). THP-1 cells were cultured in RPMI-1640 (KGL1501, KeyGEN Biotechnology, China) supplemented with 10% fetal bovine serum (FBS, 10270-106, Gibco, USA) at 37 °C and 5% CO_2_. For macrophage differentiation, THP-1 cells were seeded at 70% density in plates and treated with 70 ng/mL PMA for 24 h. After induction, cells were washed with PBS to remove residual PMA and cultured with RPMI-1640 medium for subsequent experiments.

The NCTC clone 929 (L929) cell was obtained from the China Center for Type Culture Collection and cultured in DMEM/High glucose medium (KGL1206, KeyGEN Biotechnology, China) with 10% FBS at 37 °C and 5% CO_2_. For preparation of bone marrow-derived macrophages (BMDMs) differentiation conditioned medium, L929 cells were cultured to approximately 80–90% density and maintained in complete medium for 7 days without medium replacement. The supernatant was collected, centrifuged at 3000 rpm for 10 min to remove cell debris, and filtered through a 0.22 μm membrane (SLGP033R, Merck, USA). The filtered medium was stored at −20 °C.

To collect BMDMs, C57BL/6J mice (males, 6 weeks old, GemPharmatech) were euthanized. Bone marrow cells were flushed from tibias with cold PBS and the isolated cells were cultured for 7 days in DMEM supplemented with 10% FBS, and 20% L929 conditioned medium (source of M-CSF) to differentiate into BMDMs. All cells were incubated under 5% CO_2_ at 37 °C. Fresh differentiation medium was added on day 3. After 7 days of culture, adherent cells were collected and used as BMDMs for subsequent experiments.

The THP-1 cells and BMDMs were cultured in serum-free medium for 12 h prior to treatment before the experiments. The THP-1 cells and BMDMs were incubated with ox-LDL (80 μg/mL) and treated with Nar (5 μΜ, 15 μM) simultaneously for 48 h. Meanwhile, atorvastatin (1 μM) was the positive control group. Nar and atorvastatin were dissolved in DMSO and diluted with PBS to a final DMSO concentration of 0.1%. The vehicle control (0.1% DMSO in PBS) was administered to both the control group and model group.

### Cell viability assay

The THP-1 and BMDMs were seeded into 96-well plates (NEST Biotechnology, China) at a density of 1 × 10^4^ cells/well and treated as described above. The cells were incubated with the medium containing different concentrations of Nar for 48 h. After incubating, the cells were given 10 μL CCK8 for 1 h. Finally, the absorbance was detected at 450 nm.

### Cell Oil Red staining

The THP-1 cells and the BMDMs were seeded in 24-well plates at a density of 1 × 10^5^ cells/well and treated as described above. After culturing with ox-LDL for 48 h, the THP-1 and BMDM-derived FCs were stained with 0.5 mL 0.3% Oil Red O (O0625, Sigma, USA) per well for 30 min. After staining, FCs were observed and photographed using a light microscope (Nikon, Japan).

### Intracellular lipid content determination

The THP-1 cells and the BMDMs were seeded in 24-well plates at a density of 10^5^ cells/well and treated as described above. Intracellular lipid content was measured using the TC and TG Colorimetric Assay Kit (Elabscience, China) following the manufacturer’s instructions.

### Screening of inhibitor of FCs formation

The screening of the GFs for inhibition of FCs formation was performed as follows. The THP-1 cells were seeded in 24-well plates at a suitable density. The THP-1 cells were incubated with ox-LDL and treated with GPs (30 μg/mL), GFs (30 μg/mL), GF-1 (15 μM), GF-2 (15 μM), GF-3 (15 μM), GF-4 (15 μM), GF-5 (15 μM), or Ato (1 μM) for 48 h, respectively. After treatment, Oil Red O staining, TC, and TG were performed as described above.

### Cholesterol uptake assay

The THP-1 cells and BMDMs were incubated in Dil-ox-LDL to assay cholesterol uptake. The THP-1 and BMDMs seeded in 24-well plates at a density of 10^5^ cells/well were incubated with 30 μg/mL Dil-ox-LDL and Nar (5, 15 μM) for 6 h, and the images were captured with a fluorescent microscope (Nikon, Japan).

The THP-1 cells and BMDMs were seeded in 6-well plates at a density of 3 × 10^5^ cells/well and treated as described above for the flow cytometry-based analysis of ox-LDL uptake. After Dil-ox-LDL and Nar incubation for 6 h, THP-1 cells and BMDMs were gently detached using a cell scraper and measured by Beckman flow cytometry (Excitation: 554 nm, Emission: 571 nm).

### Experimental animals and treatment

All animal experiments were approved by the Animal Research Ethics Committee of Jiangsu Academy of Traditional Chinese Medicine (Permission number: AEWC-20220401-200) and were conducted in accordance with the AVMA Guidelines for the Euthanasia of Animals. 8-weeks-old male ApoE^−/−^ mice and C57BL/6J were purchased from GemPharmatech (Nanjing, China). All mice were housed in 12-h light/dark cycles at 23 ± 1 °C and 50–70% humidity, with free access to water.

After adaptive feeding, C57BL/6J mice (*n* = 8) were assigned to the normal control group (Con). Thirty-two ApoE^−/−^ mice were stratified according to baseline body weight and randomly divided into four groups (*n* = 8 per group): Mod, Nar-L, Nar-H and Ato. Mice in the Con group were fed a control diet (10% fat, 76% carbohydrate and 14% protein, TP 26332, Trophic, China), whereas mice in the Mod, Nar-L, Nar-H, and Ato groups were fed a high-fat diet (HFD) (42% fat, 43% carbohydrate and 15% protein, 0.5% cholesterol, TP26302050, Trophic, China) for 9 weeks to induce atherosclerosis. To eliminate the confounding effect of food intake on metabolic parameters, a pair-feeding protocol was employed. The Con group, Nar-L group, Nar-H group, Ato group were pair-fed to the Mod group, receiving an equal amount of food on a daily basis based on the previous day’s consumption of the Mod group.

Narcissoside (Nar) was administered by oral gavage once daily at a dose of 10 mg/kg/day in the Nar-L group and 30 mg/kg/day in the Nar-H group for 9 weeks. Mice in the Ato group received 10 mg/kg/day Atorvastatin by oral gavage. Mice in the Con and Mod groups received an equivalent volume of 5‰ CMC-Na. The gavage volume was adjusted according to body weight (10 mL/kg). Mice were sacrificed by cervical dislocation after 9 weeks, then blood and aortas were collected.

### En face Oil Red O staining of aorta

The entire aorta was fixed in 4% paraformaldehyde (PFA) and stained with Oil Red O working solution for 20 min to visualize neutral lipid accumulation. After washing with 60% isopropanol and distilled water, images were captured using a stereomicroscope (Nikon, Japan).

### Oil Red O staining of aortic roots

Frozen sections of aortic root were cut at 6 μm thickness. Sections were fixed with 4% PFA and stained with Oil Red O working solution for 15 min. After washing with distilled water, sections were counterstained with hematoxylin. Lipid accumulation within atherosclerotic plaques was visualized under a light microscope (Nikon, Japan).

### Pathological staining of aortic roots

The aortic roots of mice were preserved in 4% PFA over 48 h for further processing that were sectioned into 5 μm slices. Hematoxylin–eosin (H&E) and Masson was conducted according to the manufacturer’s instructions (Solarbio, China). Images were captured using a light microscope (Nikon, Japan), and quantitative analysis was performed using ImageJ software.

### immunofluorescence staining of aortic roots

For immunofluorescence staining, each section of aortic roots was incubated with primary antibodies against CD68 (1:800) or α-SMA (1:600). After washing with PBS, sections were incubated with CoraLite 488 or CoraLite 594-conjugated secondary antibodies for 2 h at room temperature. Fluorescence images were captured using an inverted fluorescence microscope (Nikon, Japan).

### Levels of serum lipids

Levels of LDL-C, HDL-C, TC, and TG in serum were measured with assay kits (Elabscience, China) according to the manufacturer’s instructions.2.11 Enzyme-linked 2.14 Immunosorbent assay (ELISA).

IL-6, IL-1β, TNF-α levels were measured using commercial kits (Elabscience, China) according to the manufacturer’s instructions.

### Immunofluorescence staining of cells

The THP-1 cells and BMDMs were seeded in 6-well plates 3 × 10^5^ cells/well and treated as described above. The FCs slides were rinsed, fixed, permeabilized, blocked at room temperature, and incubated the cells overnight with Rabbit CD36 antibody (1:200) or Rabbit NR4A1 antibody (1:100). Then, we used CoraLite 488 or 594-conjugated secondary antibodies (1:100) to incubate the slides for 2 h, followed by DAPI staining for 10 min and capture images by inverted fluorescence microscope (Nikon, Japan).

### Immunohistochemistry staining

Five-micrometer-thick plaque sample sections were processed with citrate buffer (pH 6.0) at 125 °C for 3 min to antigen retrieval. Sections were incubated with anti-CD36 antibody (1:100) overnight at 4 °C after blocking with 1% BSA. Detection utilized DAB chromogen with hematoxylin counterstain (PK10006, Proteintech, China) and capture images by microscope (Nikon, Japan).

### Identification of transcription factors regulating CD36

The promoter region of CD36 was analyzed using the UCSC Genome Browser (https://genome.ucsc.edu/) and JASPAR database (https://jaspar.elixir.no/) to identify potential transcription factors. Candidates with a binding score ≥ 400 and positive transcriptional orientation (+) were selected for further screening. Atherosclerosis-related targets were retrieved from GeneCards (https://www.genecards.org/) using “atherosclerosis” as the keyword, with a relevance score ≥ 2 set as the inclusion criterion. Intersection analysis of the two datasets was performed to obtain overlapping transcription factors [16].

### ChIP-PCR

The ChIP assay was performed by BeyoChIP Enzymatic ChIP Assay Kit (P2083s, Beyotime, China) following the manufacturer’s protocol. Briefly, THP-1 cells, treated with ox-LDL and Nar (5 μM) for 48 h, were then fixed with 1% formaldehyde for 10 min, which was stopped by Glycine Solution. The cross-linked chromatin was digested with MNase and sonicated to a length of approximately 150–1000 bp. An aliquot of each ChIP sample was prepared as input control, while the rest of the DNA was incubated with anti-NR4A1 (1:100) or anti-IgG (1:100) as negative control overnight at 4 °C. After adding Protein A/G Magnetic Beads/Salmon Sperm DNA beads for 2 h, chromatin complex was washed by ChIP buffer, and decrosslinked by NaCl and proteinase K at 65 °C for 2 h. Finally, DNA purified through columns was assayed by qPCR using primers specific for the promoters of CD36. DNA enrichment was quantified by 2^[C[T]Input−Log2(Input Dilution Factor)]−C[T]ChIP^. The primer sequences were presented in Table S1.

### Luciferase reporter assay

Vectors were purchased from Yixu Biotechnology (Anhui, China). The promoter region of human CD36 containing the predicted NR4A1 binding site was cloned into the pGL3-basic luciferase reporter vector. A mutant construct containing mutations in the NR4A1 binding motif was generated using site-directed mutagenesis. CD36 promotor plasmids and NR4A1 overexpression plasmid were transfected into THP-1 cells together with the renilla luciferase plasmid pRL-TK using Lipofectamine 3000 (Invitrogen, USA) according to the manufacturer’s instructions. After 24 h of transfection, cells were treated with ox-LDL and Nar for an additional 48 h. Cells were then lysed and luciferase activity was measured using a Dual-Luciferase Reporter Assay System (DL101-01, Vazyme, China). Firefly luciferase activity was normalized to renilla luciferase activity to correct for transfection efficiency.

### RT-qPCR analysis

Total RNA was extracted from the THP-1 and BMDMs using Trizol (R411, Vazyme, China). Reverse transcription and PCR were performed with reverse transcription kits (EG15133S, Best Enzymes, China) and SYBR Green PCR Master Mix (EG20117M, Best Enzymes, China). GAPDH was used as an internal control. Data were analyzed using the method of 2^−ΔΔCt^. The sequences of PCR primers are listed in Table S1.

### Western blot analysis

Total protein and nuclear/cytoplasmic protein were respectively extracted from THP-1 or BMDMs using the Whole Cell Lysis kit and Nuclear/Cytoplasmic Protein Extraction Kit (P0028, Beyotime, China), and quantified by the BCA method (P0012, Beyotime, China). Samples were separated by SDS-PAGE and incubated at 4 °C overnight with the corresponding primary antibodies: anti-NR4A1 (1:1000), anti-CD36 (1:1000), and anti-GAPDH (1:3000), and incubated with HRP-conjugated Affinipure Goat Anti-Rabbit IgG(H + L) (1:3000) for 2 h and visualized using a commercial enhanced chemiluminescence kit (New Cell & Molecular Biotech, Suzhou, China).

### Transfection of siRNA

The siRNA of NR4A1 was purchased from Yixu Biotechnology (Anhui, China). The THP-1 cells and BMDMs were seeded in 6-well plates 3 × 10^5^ cells/well. The 50 nmol/L siRNAs were transfected into cells using Lipofectamine 3000. After 6 h of incubation, the medium was replaced with fresh complete medium. Cells were harvested 24–48 h after transfection for subsequent analyses. The knockdown efficiency of NR4A1 was verified by RT-qPCR and Western blot analysis. The sequences of si-*NR4A1* are listed in Table S1.

### Statistical analysis

Data normality was assessed using the Kolmogorov–Smirnov test. For normally distributed data, differences among multiple groups were analyzed using one-way ANOVA followed by Tukey’s post-hoc test, whereas non-normally distributed data were analyzed using the Kruskal–Wallis test. For comparisons between two groups, the unpaired Student’s *t* test or Mann–Whitney *U* test was applied as appropriate. All data are presented as the mean ± SD. The *P* value <0.05 was considered statistically significant.

## Results

### Narcissoside attenuates lipid accumulation in THP-1 cells and BMDMs

Since FCs are key contributors in AS, the potential inhibitory effects of GFs and GPs were examined using ox-LDL-induced THP-1 cells as an in vitro model. Ox-LDL (80 μg/mL for 48 h) successfully induced FCs formation, and GFs (30 μg/mL) exhibited a superior inhibitory effect on the FCs formation compared to GPs (30 μg/mL) (Fig. S2). To further identify the active constituents in GFs, this fraction was purified by preparative HPLC, leading to the isolation of five flavonoids. Among them, GF-4 (Narcissoside) exhibited a significant inhibitory effect on lipid accumulation (Fig. S2). Therefore, Narcissoside (Nar) was selected as the lead compound for subsequent studies on FCs formation.

**Fig. 1:**
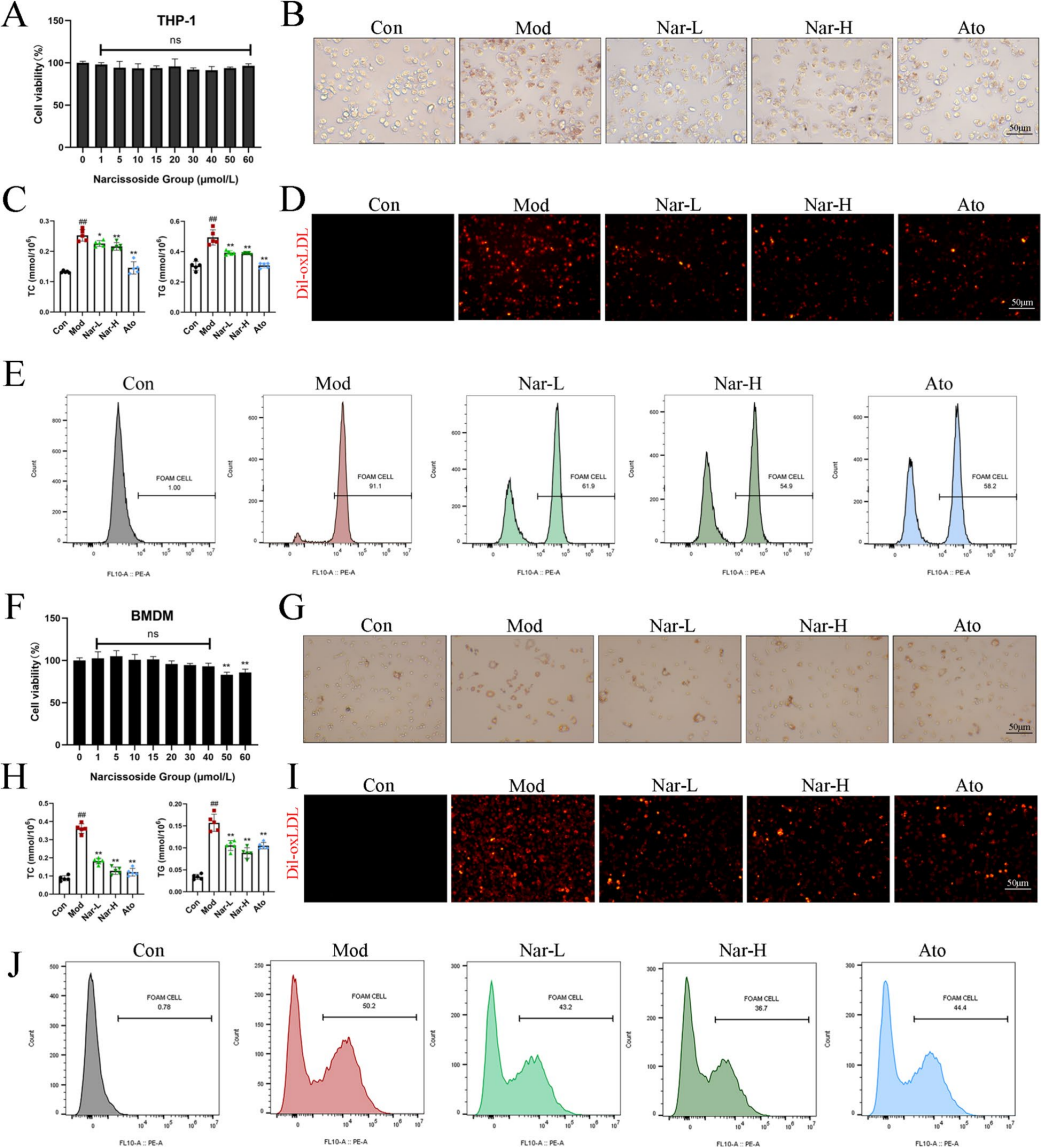
Narcissoside attenuates lipid accumulation in THP-1 cells and BMDMs. **A** The viability of BMDMs after treated with Nar (1, 5, 10, 15, 20, 30, 40, 50 and 60 μM) for 48 h was tested using CCK-8 kit. **B** THP-1 cells were treated with 80 μg/mL ox-LDL in the presence or absence of Nar (5 and 15 μM) or Ato (1 μM) for 48 h. The lipid droplet formation was determined by Oil Red O staining. Scale bar = 50 μm. **C** Comparison of THP-1 cells total cholesterol (TC) and triglycerides (TG). **D** Fluorescent images for macrophage lipid uptake in THP-1 cells. THP-1 cells were stained with Dil-ox-LDL (30 μg/mL) after treatment with Nar (5 and 15 μM) or ATO (1 μM) for 6 h. Scale bar = 50 μm. **E** Cholesterol uptake assessed by flow cytometer in THP-1 cells. **F** The viability of BMDMs after treated with Nar (1, 5, 10, 15, 20, 30, 40, 50 and 60 μM) for 48 h was tested using CCK-8 kit **G** BMDMs were treated with 80 μg/mL ox-LDL in the presence or absence of Nar (5 and 15 μM) or ATO (1 μM) for 48 h. The lipid droplet formation was determined by Oil Red O staining. Scale bar = 50 μm. **H** Comparison of BMDMs total cholesterol (TC) and triglycerides (TG). **I** Fluorescent images for macrophage lipid uptake in BMDMs. BMDMs was stained with Dil-ox-LDL (30 μg/mL) after treatment with Nar (5 and 15 μM) or ATO (1 μM) for 6 h. Scale bar = 50 μm. **J** Cholesterol uptake assessed by flow cytometer in BMDMs. Data are expressed as mean ± SD; *n* = 3 for each group ^#^ *P* < 0.05, ^##^ *P* < 0.01 vs. Con group, * *P* < 0.05, ** *P* < 0.01 vs. Mod group

To assess the potential cytotoxicity of Nar, THP-1 cells were treated with Nar at concentrations ranging from 0 to 60 µM (Fig. 1A). The results showed that Nar exhibited low cytotoxicity toward macrophages at concentrations below 60 μM for 48 h. Therefore, 5 μM and 15 μM Nar were chosen in the subsequent experiments. As shown in Fig. 1B, Nar significantly decreased the FCs formation and attenuated lipid accumulation (Fig. 1B). Quantitative analysis of TC and TG in THP-1 cells revealed a similar trend, with Nar effectively reducing intracellular lipid accumulation (Fig. 1C). To further assess cholesterol uptake of Nar, THP-1 cells were incubated with Dil-ox-LDL for 6 h. The data showed that intense staining was observed in the Mod group, whereas Nar treatment markedly reduced fluorescence intensity (Fig. 1D). Flow cytometric analysis showed that treatment with 15 μM Nar reduced cholesterol uptake by approximately 45% compared with the Mod group (Fig. 1E). Importantly, the effect of Nar on inhibiting FCs formation was not restricted to THP-1 cells. Consistently, Nar also decreased intracellular lipid accumulation and cholesterol uptake in ox-LDL-stimulated BMDMs for 48 h (Fig. 1F–J). Collectively, these results indicated that Nar has the potential to diminish the formation of FCs and lipid accumulation.

### Narcissoside inhibits plaque formation and lipid deposition in ApoE^−/−^ mice

**Fig. 2:**
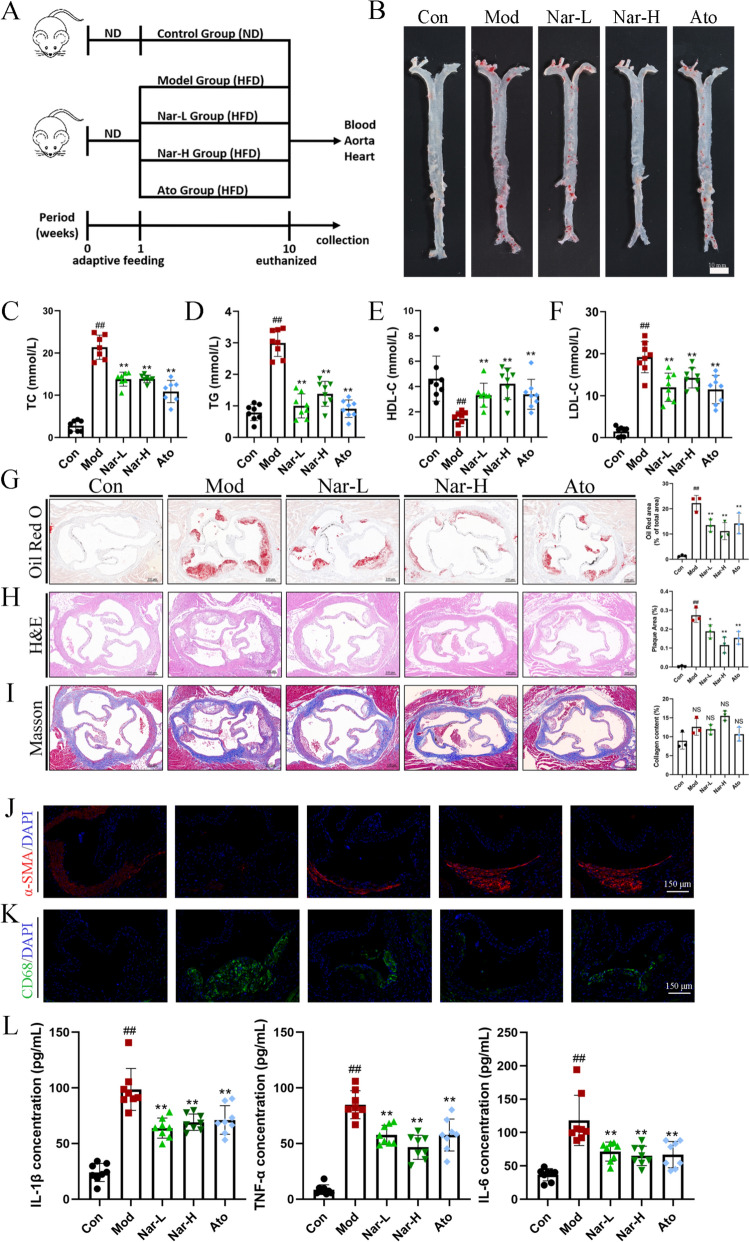
Narcissoside inhibits plaque formation and lipid accumulation in ApoE^−/−^ mice. **A** Experiment outline. ApoE^−/−^ mice was treated with Nar (10 and 30 mg/kg, daily by oral gavage) or Ato (10 mg/kg, daily by oral gavage) for 9 weeks **B** After treatment, aortas were isolated for determination of lesions in en face aortas by Oil Red O staining (*n* = 3 per group). Scale bar = 10 mm. Serum TC level (**C**), TG level (**D**), LDL-C level (**E**), HDL-C level (**F**) (*n* = 8 per group). Representative photomicrographs (left) and quantification (right) of aortic root sections stained with Oil Red O (**G**), H&E (**H**), and Masson (**I**) in atherosclerotic plaque (*n* = 3 per group). Scale bar = 100 μm. **J** Representative photomicrographs of aortic root sections stained with CD68 in atherosclerotic plaque (*n* = 3 per group). Scale bar = 150 μm. **K** Representative photomicrographs of aortic root sections stained with α-SMA in atherosclerotic plaque (*n* = 3 per group). Scale bar = 150 μm. **L** The serum inflammatory cytokine expression of IL-1β, IL-6, and TNF-α (*n* = 8 per group). Data are expressed as mean ± SD. ^#^ *P* < 0.05, ^##^ *P* < 0.01 vs. Con group, * *P* < 0.05, ** *P* < 0.01 vs. Mod group

To investigate the therapeutic effects of Nar on AS, ApoE^−/−^ mice were administered Nar (10 or 30 mg/kg/day) or atorvastatin (Ato, 10 mg/kg/day) by intragastric gavage for 9 weeks (Fig. 2A). En face Oil Red O staining of the entire aorta showed that Nar markedly reduced lipid-rich plaque area compared with the Mod group (Fig. 2B). The serum levels of TC, TG and LDL-C were significantly increased and the HDL-C level was decreased in the Mod group compared with the Con group, whereas Nar treatment dose-dependently alleviated these lipid levels (Fig. 2C–F). Furthermore, histological analysis showed that the Mod group exhibited larger necrotic cores and greater lipid deposition in the aortic root compared with the Con group. In contrast to the Mod group, Nar treatment resulted in a reduction of necrotic core and lipid deposition within aortic plaques (Fig. 2G, H). However, there was no significant difference in the collagen content among all groups (Fig. 2I). Immunofluorescence staining of the aortic root revealed that the fluorescence intensity of CD68, a marker for macrophages, was significantly elevated in the Mod group compared to the Con group, indicating an increased macrophage infiltration. Conversely, treatment with Nar markedly reduced CD68 fluorescence (Fig. 2J). In contrast, the expression of α-SMA, a marker for vascular smooth muscle cells, showed an opposing trend. There was a notable reduction in α-SMA-positive cells from Mod group, which was effectively reversed upon Nar administration (Fig. 2K). Given the significant role of inflammation in AS, the impact of Nar on the inflammatory response was detected in the serum of ApoE^−/−^ mice. Serum levels of IL-1β, IL-6, and TNF-α was found to be upregulated in the Mod group, while Nar treatment effectively reversed this uptrend (Fig. 2L). Taken together, Nar attenuates the development of AS and decreases serum inflammation.

### Narcissoside attenuates ox-LDL uptake in THP-1 cells and BMDMs

**Fig. 3:**
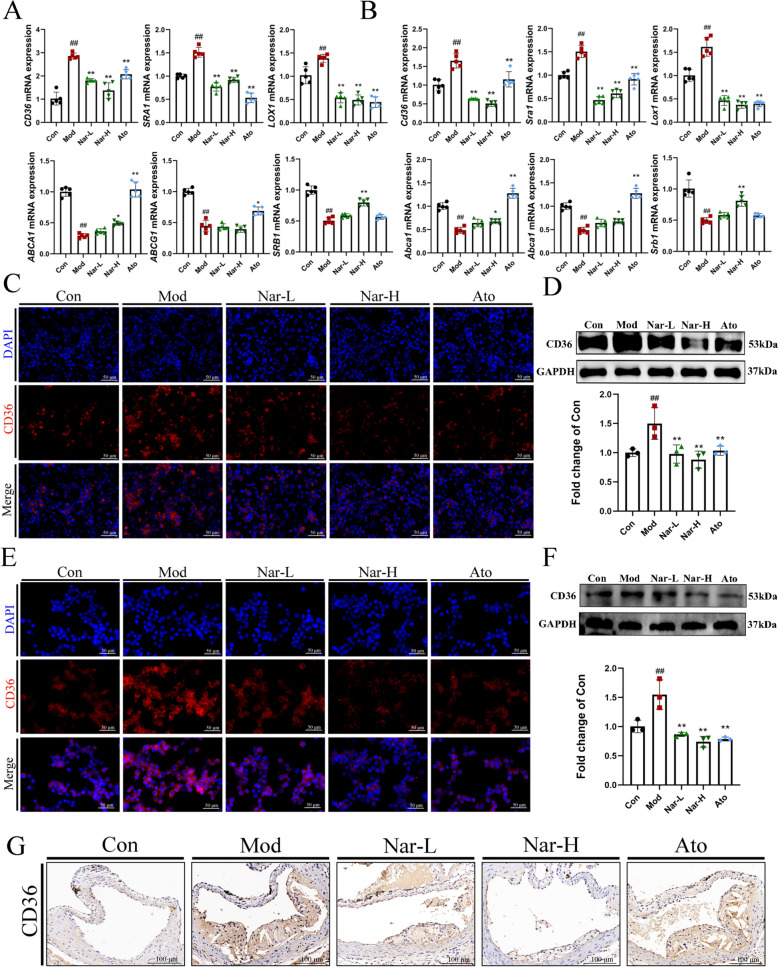
Narcissoside attenuates ox-LDL uptake in THP-1 cells or BMDMs. **A**,**B** The relative mRNA expression of macrophage cholesterol uptake (*CD36*, *SRA1*, *LOX1*) and efflux (*ABCA1*, *ABCG1*, *SRB1*) in THP-1 cells or BMDMs using RT-qPCR (*n* = 5 per group). **C** The relative protein expression of CD36 by immunofluorescence staining in THP-1 (*n* = 3 per group). Scale bar = 50 μm. **D** The relative protein expression of CD36 by Western blot in THP-1 cells and quantification of protein. **E** The relative protein expression of CD36 by immunofluorescence staining in BMDMs (*n* = 3 per group). Scale bar = 50 μm. **F** The relative protein expression of CD36 by Western blot in BMDM and quantification of protein. **G** Representative images of CD36 staining of aortic root sections by immunohistochemistry staining (*n* = 3 per group). Scale bar = 150 μm. Data are expressed as mean ± SD. ^#^ *P* < 0.05, ^##^ *P* < 0.01 vs. Con group, * *P* < 0.05, ** *P* < 0.01 vs. Mod group

In FCs, the imbalance between the uptake and efflux of cholesterol, which is regulated by scavenger receptors and cholesterol transporters, is a primary factor contributing to their formation. Consequently, the mRNA expression levels of scavenger receptors (*SRA1*, *CD36*, *LOX1*) and cholesterol transporters (*SRB1*, *ABCA1*, *ABCG1*) by RT-qPCR. Nar significantly suppressed the mRNA levels of *SRA1*, *CD36*, and *LOX1*, whereas no significant changes were observed in the expression of cholesterol transporters (Fig. 3A, B). Given that CD36 accounts for approximately 70% of ox-LDL uptake, CD36 expression was further examined by immunofluorescence and western blot analysis, and Nar treatment markedly decreased CD36 expression (Fig. 3C–F). Additionally, immunohistochemical staining of aortic root plaques using anti-CD36 antibodies revealed that Nar treatment reduced the CD36-positive area within plaques (Fig. 3G). These findings suggested that Nar alleviated FCs generation by downregulating the expression of CD36.

### Narcissoside inhibits CD36 expression by increasing NR4A1 expression and promoting nuclear translocation

**Fig. 4:**
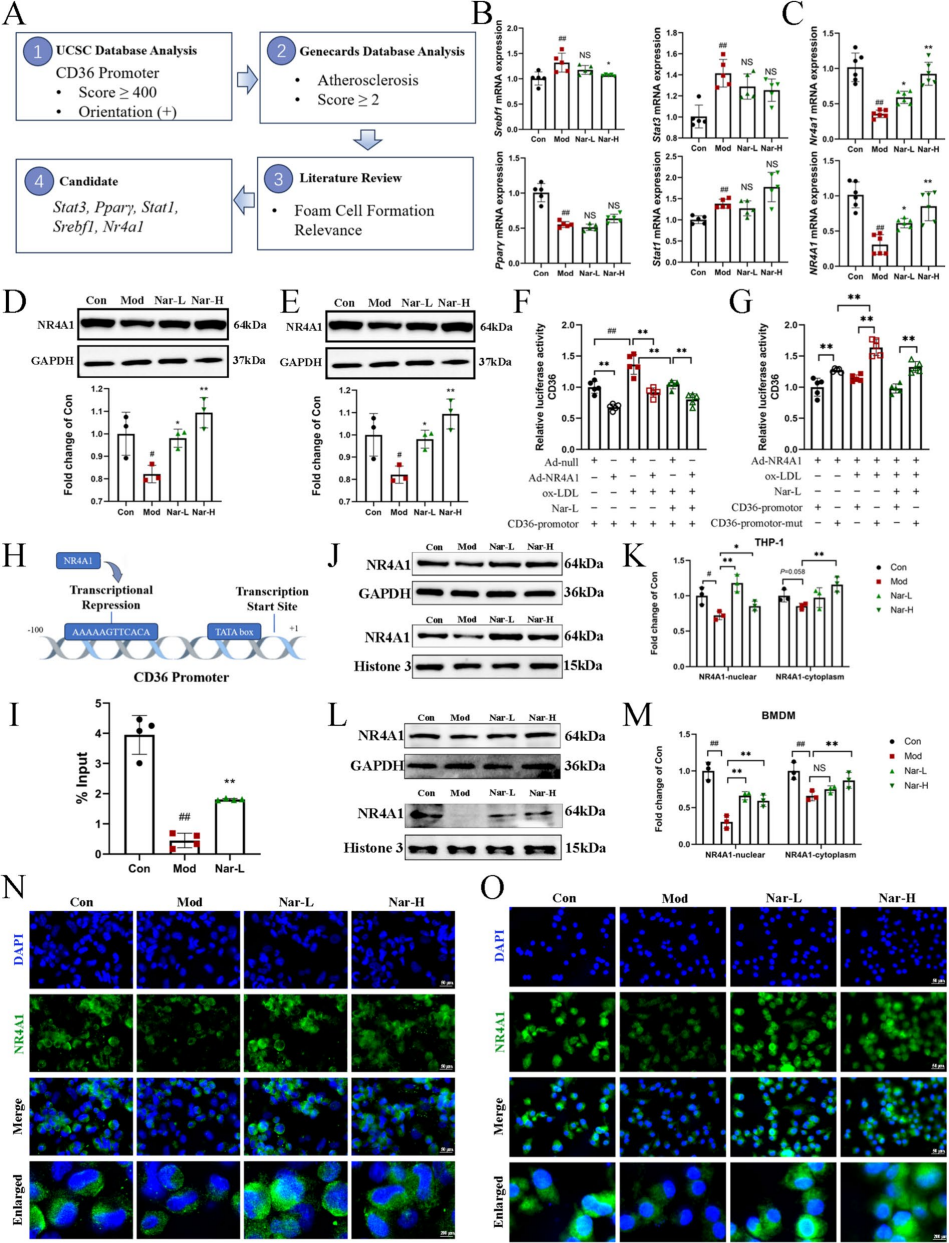
Narcissoside reduces the formation of FCs by enhancing NR4A1 expression and promoting nuclear translocation. **A** Experiment outline. CD36 potential transcription factors were retrieved from the UCSC database. Atherosclerosis-related genes were obtained from the GeneCards database. Overlapping candidates were identified by Venn diagram analysis. List of five transcription factors (*Pparg*, *Stat3*, *Stat1*, *Nr4a1*, *Srebf1*) that were ultimately selected based on literature curation for their documented roles in foam cell formation. **B** The mRNA of *Pparg*, *Stat3*, *Stat1*, *Foxp3* in BMDMs using RT-qPCR (*n* = 5 per group). **C** The relative mRNA expression of *Nr4a1* in BMDMs (up) and THP-1 cells (down) (*n* = 5 per group). **D** The relative protein expression of NR4A1 in THP-1 cells and quantification of protein (*n* = 3 per group). **E** The relative protein expression of NR4A1 in BMDMs and quantification of protein (*n* = 3 per group). **F** Effect of NR4A1 and Nar on transcription activities of wild-type in CD36 promoters detected by luciferase assay (*n* = 5 per group). **G** Effect of NR4A1 and Nar on transcription activities of mutations of the CD36 promoter AAAAAGTTCACA region, measured by luciferase assay (*n* = 5 per group). **H** Schematic diagram of the *CD36* promoter and the predicted transcription factor binding site. **I** ChIP-PCR analysis of the *CD36* promoters using antibodies against NR4A1 in ox-LDL-induced THP-1 cells (*n* = 4 per group). **J** The relative nuclear and cytoplasmic protein expression of NR4A1 in THP-1 cells (*n* = 3 per group). **K** Quantification of Fig. 5J. Data were presented as relative quantities after normalization to GAPDH or Histone 3. **L** The relative nuclear protein and cytoplasmic protein expression of NR4A1 in BMDMs (*n* = 3 per group). **M** Quantification of Fig. 5L. Data were presented as relative quantities after being normalized to GAPDH or Histone 3. **N**,**O** The relative protein expression and nuclear translocation of NR4A1 by immunofluorescence staining in THP-1 cells and BMDMs (*n* = 3 per group). Scale bar = 50 μm; enlarged scale bar = 200 μm. Data are expressed as mean ± SD. ^#^ *P* < 0.05, ^##^ *P* < 0.01 vs. Con group, * *P* < 0.05, ** *P* < 0.01 vs. Mod group

To further elucidate the mechanism by which Nar regulates CD36 expression, the UCSC database was queried to identify potential transcription factors associated with the CD36 promoter. Candidates with a score ≥ 400 and a positive transcriptional orientation (+) were selected for further analysis. Concurrently, the GeneCards database was used to retrieve atherosclerosis-related targets using a relevance score ≥ 2 as the inclusion criterion, resulting in 1165 candidate genes. Intersection analysis of the two datasets identified 17 transcription factors. Subsequently, a comprehensive literature review was performed to evaluate the association between these 17 transcription factors and FCs formation. Based on the strength of evidence and biological relevance, *Stat3*, *Pparγ*, *Stat1*, *Nr4a1*, and *Srebf1* were selected for further investigation (Fig. 4A). Ox-LDL stimulation resulted in a marked reduction in *Nr4a1* mRNA expression, which was subsequently restored after Nar treatment (Fig. 4B, C). Western blot analysis revealed that Nar treatment significantly increased NR4A1 protein expression in THP-1 cells and BMDMs (Fig. 4D, E). To ascertain whether the suppression of CD36 occurs at the transcriptional level, CD36 promoter plasmids were constructed. NR4A1 overexpression significantly decreased CD36 promoter activity, indicating that NR4A1 negatively regulates CD36 expression, particularly in the presence of ox-LDL. Notably, Nar treatment enhanced the transcriptional suppression of CD36 mediated by NR4A1 (Fig. 4F). UCSC and JASPAR database sequence analysis identified two putative NR4A1 response elements within the CD36 promoter region, of which only AAAAAGTTCACA was located in the same transcriptional orientation as CD36 (Fig. S3A, B). Therefore, a mutant CD36 promoter was constructed with a mutation of AAAAAGTTCACA. The activity of the mutated CD36 promoter was significantly upregulated compared to the wild-type CD36 promoter, further suggesting that NR4A1 may inhibit CD36 expression by binding to the NR4A1 response element. Meanwhile, the inhibitory effect of Nar on CD36 promoter activity was significantly attenuated upon mutation of the NR4A1 binding site (Fig. 4G). Consistently, quantitative chromatin immunoprecipitation (ChIP) analysis indicated that NR4A1 enrichment on *CD36* promoters were significantly decreased in ox-LDL-induced THP-1 cells, which was elevated with the treatment of Nar (Fig. 4H, I). These results imply that Nar suppressed the CD36 expression by enhancing NR4A1-mediated transcriptional repression on the NR4A1 response element region of the CD36 promoter.

As a transcription factor, the subcellular localization of NR4A1 is crucial for its functional activity. Therefore, the impact of Nar on NR4A1 nuclear translocation was investigated. In FCs, NR4A1 was predominantly excluded from the nucleus. The results showed that NR4A1 expression increased in both the nucleus and cytoplasm after Nar treatment, with a particularly pronounced elevation observed in the nucleus (Fig. 4J–M). To investigate the effect of Nar on NR4A1 nuclear translocation, NR4A1 nuclear translocation was assessed by immunofluorescence. The results demonstrated that Nar promoted the nuclear translocation of NR4A1, thereby enhancing its role as a transcription factor (Fig. 4N, O). These findings suggest that Nar inhibits CD36 expression by increasing NR4A1 expression and promoting its nuclear translocation.

### Silencing of NR4A1 promotes the formation of FCs

**Fig. 5:**
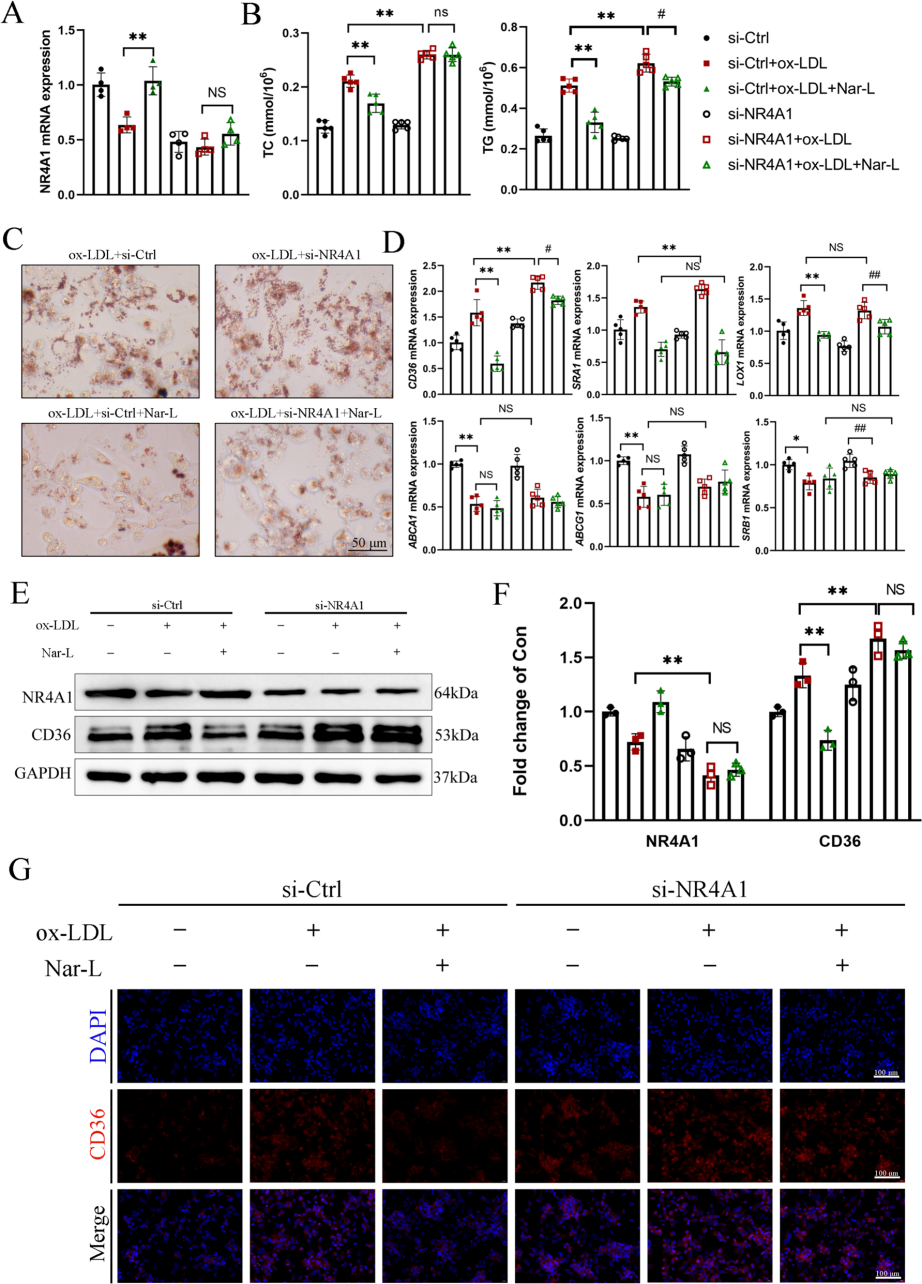
Knocking down NR4A1 accelerates the formation of FCs in THP-1 cells. **A** The relative mRNA expression of *NR4A1* after si-*NR4A1* treatment in THP-1 (*n* = 4 per group). **B** Comparison of THP-1 cells total cholesterol (TC) and triglycerides (TG) after si-*NR4A1* treatment (*n* = 5 per group). **C** THP-1 cells were stained with Oil Red O after si-*NR4A1* treatment (*n* = 3 per group). Scale bar = 50 μm. **D** The relative mRNA expression of macrophage cholesterol uptake (*CD36*, *SRA1*, *LOX1*) and efflux (*ABCA1*, *ABCG1*, *SRB1*) in THP-1 cells after si-*NR4A1* treatment (*n* = 5 per group). **E** The relative protein expression of CD36 and NR4A1 by Western blot in THP-1 cells (*n* = 3 per group). **F** Quantification of **E**. Data were presented as relative quantities after being normalized to GAPDH. **G** The relative protein expression of CD36 by immunofluorescence in THP-1 after si-*NR4A1* treatment (*n* = 3 per group). Scale bar = 100 μm. Data are expressed as mean ± SD. ^#^ *P* < 0.05, ^##^ *P* < 0.01 vs. si-NR4A1 + ox-LDL, * *P* < 0.05, ** *P* < 0.01 vs. si-Ctrl + ox-LDL

To investigate the role of NR4A1 in FCs, a siRNA-mediated knockdown of NR4A1 was performed (Fig. 5A). NR4A1 knockdown significantly increased TC and TG contents in FCs, while substantially attenuating the effect of Nar in TC and TG (Fig. 5B). Oil Red O staining showed that the knockdown of NR4A1 significantly promoted lipid accumulation in FCs (Fig. 5C). Furthermore, the NR4A1 knockdown counteracted the effects of Nar in ox-LDL-induced lipid accumulation (Fig. 5C). Notable increases were observed in the mRNA levels of *CD36* and *SRA1* following the knockdown, whereas the expression of other genes remained unchanged (Fig. 5D). To validate the impact of NR4A1 knockdown on CD36, western blot and immunofluorescence analyses were performed. Nar treatment reduced CD36 expression, which was markedly reversed by NR4A1 silencing (Fig. 5E, F).

**Fig. 6:**
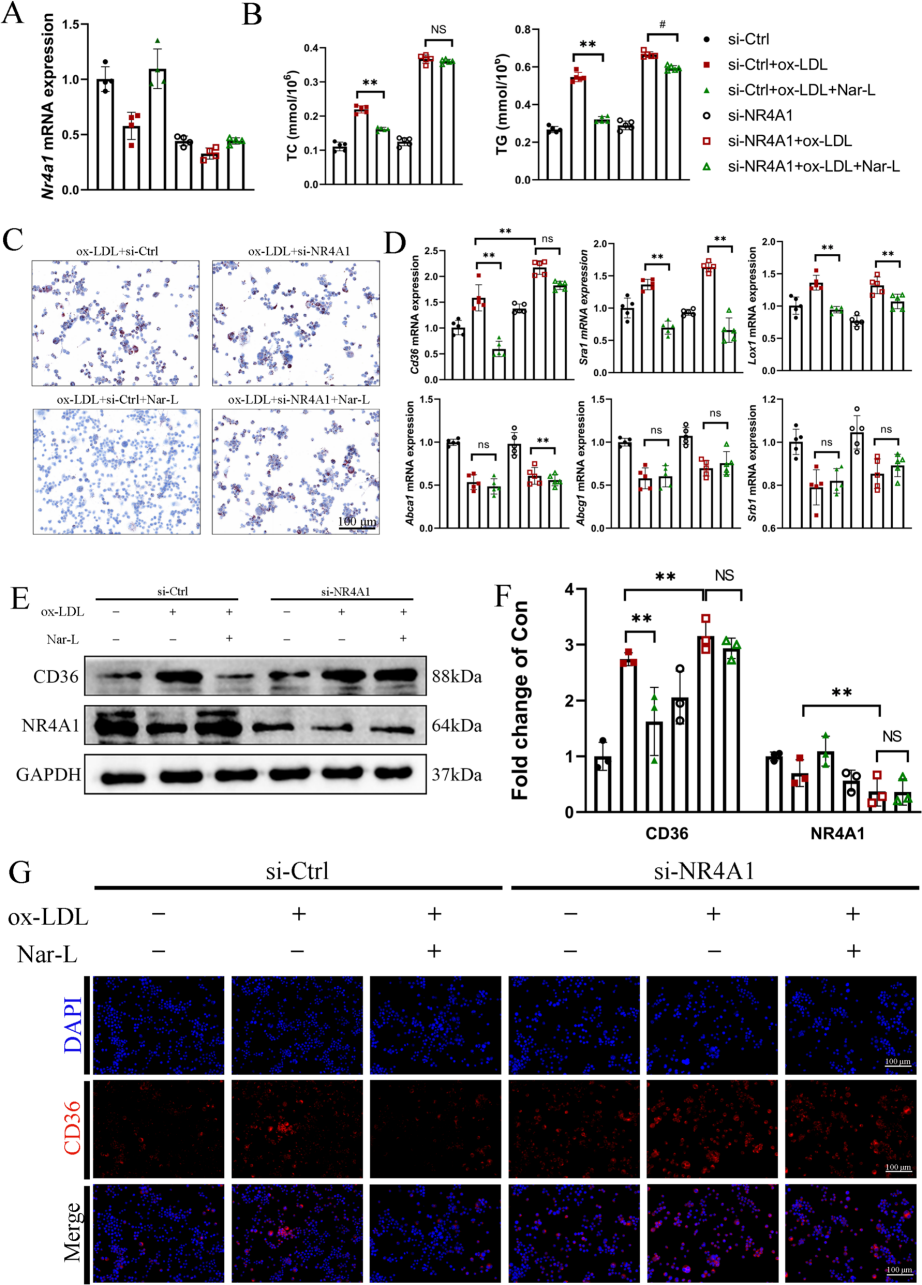
Knocking down NR4A1 accelerates the formation of FCs in BMDMs. **A** The relative mRNA expression of *Nr4a1* after si-*Nr4a1* treatment in BMDMs (*n* = 4 per group). **B** Comparison of BMDMs total cholesterol (TC) and triglycerides (TG) after si-*Nr4a1* treatment (*n* = 5 per group). **C** BMDMs were stained with Oil Red O after si-*Nr4a1* treatment (*n* = 3 per group). Scale bar = 100 μm. **D** The relative mRNA expression of macrophage cholesterol uptake (*Cd36*, *Sra1*, *Lox1*) and efflux (*Abca1*, *Abcg1*, *Srb1*) in BMDMs after si-*Nr4a1* treatment (*n* = 5 per group). **E** The relative protein expression of CD36 and NR4A1 by Western blot in BMDMs (*n* = 3 per group). **F** Quantification of **E**. Data were presented as relative quantities after being normalized to GAPDH. **G** The relative protein expression of CD36 by immunofluorescence in BMDMs after si-*Nr4a1* treatment (*n* = 3 per group). Scale bar = 100 μm. Data are expressed as mean ± SD. ^#^ *P* < 0.05, ^##^ *P* < 0.01 vs. si-NR4A1 + ox-LDL, * *P* < 0.05, ** *P* < 0.01 vs. si-Ctrl + ox-LDL

To further exclude cell line-specific effects of our findings, BMDMs were employed to validate the role of NR4A1. The knockdown efficiency of NR4A1 in BMDMs was confirmed by RT-qPCR showing a significant reduction in NR4A1 mRNA expression (Fig. 6A). Consistent with the observations in THP-1 cells, Nar significantly reduced ox-LDL-induced lipid accumulation in BMDMs, whereas NR4A1 deficiency markedly attenuated lipid-lowering effect (Fig. 6B, C). Moreover, silencing of NR4A1 abolished the suppressive effect of Nar on CD36 protein expression, as demonstrated by Western blot and immunofluorescence analyses (Fig. 6D–G). Collectively, these data suggested that Nar may enhance NR4A1 expression, thereby inhibiting CD36-mediated FCs formation and alleviating lipid accumulation.

## Discussion

In the present study, Nar was screened as the most effective compound in inhibiting the FCs formation among the flavonoids derived from *G. pentaphyllum*. Our data revealed that Nar significantly improved the lipid profile, reduced vascular inflammation, and attenuated plaque formation in ApoE^−/−^ mice. Furthermore, Nar effectively decreased the formation of FCs in both BMDMs and THP-1 cells by inhibiting ox-LDL uptake. Mechanistically, Nar promoted NR4A1 expression and nuclear translocation, which subsequently inhibited CD36-mediated ox-LDL uptake and reduced intracellular lipid accumulation. These findings provide initial evidence for the beneficial role of Nar as a potential AS therapeutic candidate and offer new perspectives for AS treatment.

Narcissoside (Nar) is a naturally occurring flavonol glycoside widely distributed in medicinal plants such as *Bupleurum chinense* DC, *Hippophaë rhamnoides* L., and *Sambucus nigra*. Accumulating evidence indicates that Nar exhibits diverse pharmacological activities across multiple cellular and molecular pathways. For instance, Nar has been shown to increase glutathione levels, and attenuate neurotoxins-induced apoptosis by enhancing the miR-200a/Nrf2/GSH antioxidant pathway [16, 17]. Beyond neuroprotection, recent work suggests that Nar exerts antidepressant-like effects by suppressing neuroinflammation and promoting adult hippocampal neurogenesis via activation of the TREM2/PI3K/Akt pathway [18]. Additionally, Nar displays anti-inflammatory activity by suppressing pro-inflammatory cytokine production and attenuating inflammatory signaling cascades in macrophages [19]. Recent investigations further suggest potential roles in neuroprotection, metabolic enzyme modulation [20], and ion channel regulation, highlighting its pleiotropic biological functions. Notably, these pharmacological features-particularly the ability to simultaneously regulate oxidative stress and inflammation-are highly relevant to the pathogenesis of atherosclerosis. In this context, our findings extend the current understanding of Nar by identifying NR4A1-mediated regulation of CD36 as a novel mechanism contributing to its vascular protective effects.

Despite being a first-line therapy for the clinical management of AS, atorvastatin is associated with several adverse reactions, including liver dysfunction and myopathy after long-term use [21, 22]. The coadministration of atorvastatin with colchicine (another FDA-approved drug for AS treatment) may exacerbate these adverse effects, potentially due to the concurrent metabolism of both drugs via the CYP3A4 pathway [23–25]. In our research, we utilized ProTox-3.0 database to compare the toxicity of Nar with atorvastatin, which revealed superior safety profile compared with atorvastatin (Fig. S3A). Furthermore, comparative analysis in ADMElab 3.0 database suggested that the metabolism of Nar may not involve CYP3A4 pathway (Fig. S3B), and the anti-AS activity of Nar was comparable to atorvastatin. These findings suggest that Nar may represent a safe and efficacious candidate compound combined with colchicine for the clinical treatment of AS.

In addition to lipids, chronic inflammatory activity in blood vessels triggers major adverse cardiovascular events (MACE) and serves as another independent risk factor for AS [26]. Meanwhile, the accumulation of lipids in the vascular intima was reported as a crucial contributor to vascular inflammation. This increase in vascular inflammation exacerbates endothelial dysfunction, thereby facilitating lipid deposition. The addition of colchicine to lipid-lowering therapies has proven to further decrease the incidence of MACE [27]. Therefore, lipids and vascular inflammation were considered as the predominant processes in the development of AS, highlighting an urgent clinical need for synergistic therapeutic approaches. Previous research showed that CD36 plays a critical role in both lipid metabolism and inflammatory processes. The upregulation of CD36 leads to abnormal actin polymerization in macrophages, ultimately resulting in lipid accumulation within blood vessels [28]. The interaction between CD36 and toll-like receptor 4 (TLR4) activates NLRP3-mediated inflammatory responses and the NF-κB signaling pathway in macrophages [29, 30]. Recent studies have indicated that CD36 drives vascular inflammation through mitochondrial metabolism reprogramming [31]. Overall, CD36 serves as a crucial mediator linking inflammation and lipid metabolism, playing a central role in both processes. In our research, Nar significantly inhibited CD36-mediated lipid uptake in FCs and serum inflammation. Taken together, Nar may exert a synergistic effect on lipid accumulation and vascular inflammation by inhibiting the expression of CD36.

Of particular note, the precise role of NR4A1 in FCs formation remains to be fully elucidated. Previous studies using NR4A1^−/−^ LDLR^−/−^ Ly6C^+^ monocytes or NR4A1-deficient macrophages reported no significant inhibition of ox-LDL uptake or CD36 expression [32]. In contrast, other investigations employing siRNA-mediated knockdown of NR4A1 in THP-1 cell-derived macrophages demonstrated enhanced ox-LDL uptake and increased CD36 expression following NR4A1 suppression [33, 34]. These seemingly conflicting findings may be attributed to substantial methodological differences across studies. Firstly, experimental conditions such as ox-LDL concentration and incubation duration are critical determinants of lipid uptake. It has been reported that the suppressive effect of NR4A1 on ox-LDL uptake becomes more pronounced with prolonged exposure [35]. In our study, macrophages were incubated with ox-LDL for 48 h, whereas some previous studies used a 24 h exposure time. Secondly, CD36 expression is closely associated with differentiation status. Huh et al. demonstrated that CD36 is markedly upregulated during monocyte-to-macrophage differentiation [35], suggesting that baseline CD36 expression levels and differentiation context may determine whether NR4A1 functions predominantly as a transcriptional suppressor or exerts minimal observable effect. Moreover, differences in genetic manipulation strategies may contribute to the observed discrepancies. Whole-body knockout models may induce compensatory transcriptional adaptations during development, potentially masking the direct regulatory effects of NR4A1 on CD36. In contrast, acute knockdown approaches in cultured macrophages may more faithfully reflect direct transcriptional regulation without such compensation mechanism. In the present study, NR4A1 was found to directly bind to the AAAAAGTTCACA motif within the CD36 promoter and transcriptionally represses CD36 expression under ox-LDL stimulation. These findings support a model in which NR4A1 acts as a negative regulator of lipid uptake in differentiated macrophages during sustained atherogenic stress. Overall, NR4A1 may present a crucial factor in inhibiting FCs formation. Further investigation is warranted to elucidate the mechanisms underlying the context-specific regulatory role of NR4A1 in macrophage lipid metabolism.

Collectively, Nar significantly inhibited CD36 transcription by upregulating NR4A1 expression, thereby reducing FCs formation and alleviating AS. However, several limitations should be noted. First, it is essential to utilize myeloid-specific NR4A1 knockout ApoE^−/−^ mice to validate the therapeutic efficacy of Nar in attenuating AS. Additionally, future research will explore the potential intracellular interaction between Nar and NR4A1, as well as the specific binding sites of NR4A1.

## Conclusion

This study not only confirms that Nar is the active component of GFs in inhibiting FCs formation, but it also underscores its significant therapeutic effect on the uptake of ox-LDL by activating the NR4A1/CD36 pathways. These findings provide a novel direction for further investigation and potential application of Nar for AS treatment.

## Supplementary Information

Additional file 1.

## Data Availability

No datasets were generated or analysed during the current study.

## Abbreviations

ApoE: Apolipoprotein E
ASCVD: Atherosclerotic cardiovascular disease
CD36: Cluster of differentiation 36
CVD: Cardiovascular disease
FCs: Foam cells
GFs: *Gynostemma pentaphyllum* Flavonoids
GPs: Gypenosides
HDL-C: High-density lipoprotein cholesterol
H&E: Hematoxylin-eosin
HFD: High fat diet
IL-6: Interleukin-6
IL-1β: Interleukin-1β
M-CSF: Macrophage colony-stimulating factor
Nar: Narcissoside
NR4A1: Nuclear receptor subfamily 4 group A member 1
ox-LDL: Oxidized low-density lipoprotein
SR-A: Scavenger receptor class A
SR-B1: Scavenger receptor class B type 1
TC: Total cholesterol
TG: Total triglyceride
TNF-α: Tumor necrosis factor-α
