# Supplementary material for: Narcissoside attenuates atherosclerosis by suppressing CD36-mediated foam cell formation via upregulation of NR4A1
Source: Chin Med. 2026 May 20;21:136. doi: 10.1186/s13020-026-01412-1 (PMC13188464; doi:10.1186/s13020-026-01412-1)
Supplement: Supplementary file 1 — Additional file 1. [file 13020_2026_1412_MOESM1_ESM.docx]

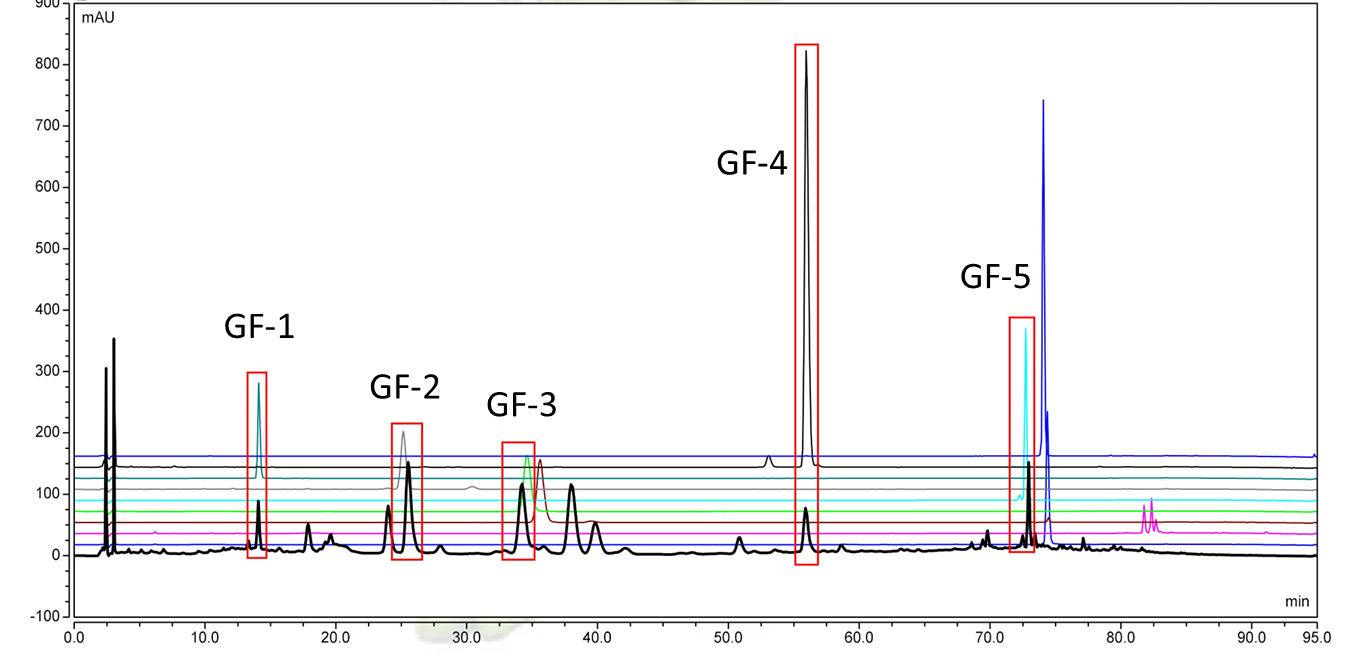


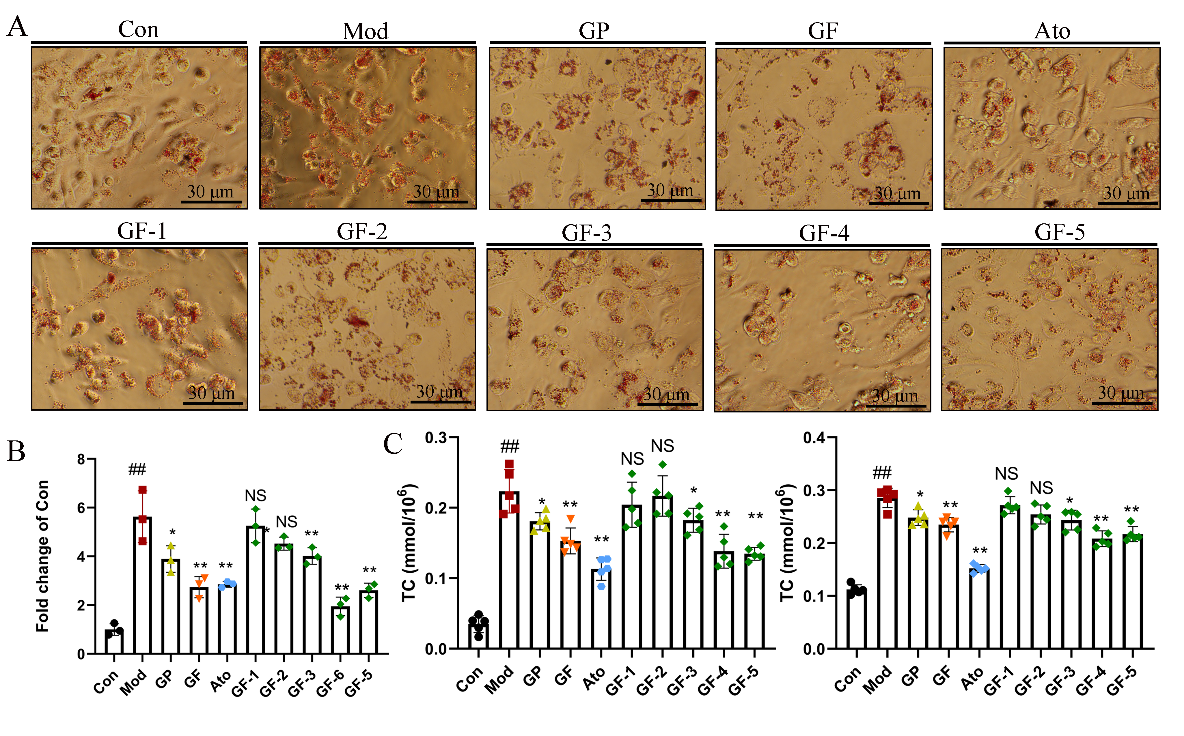


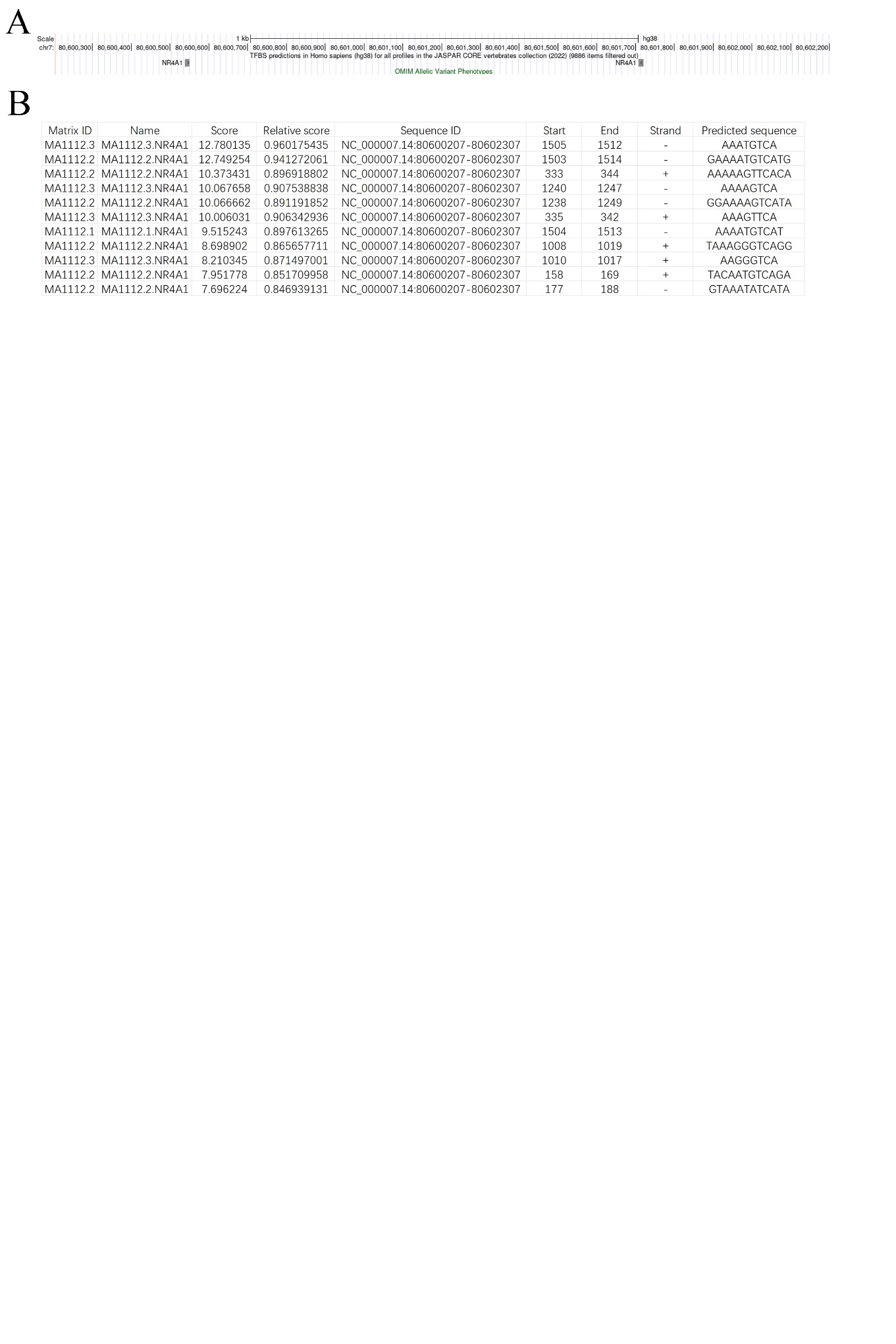


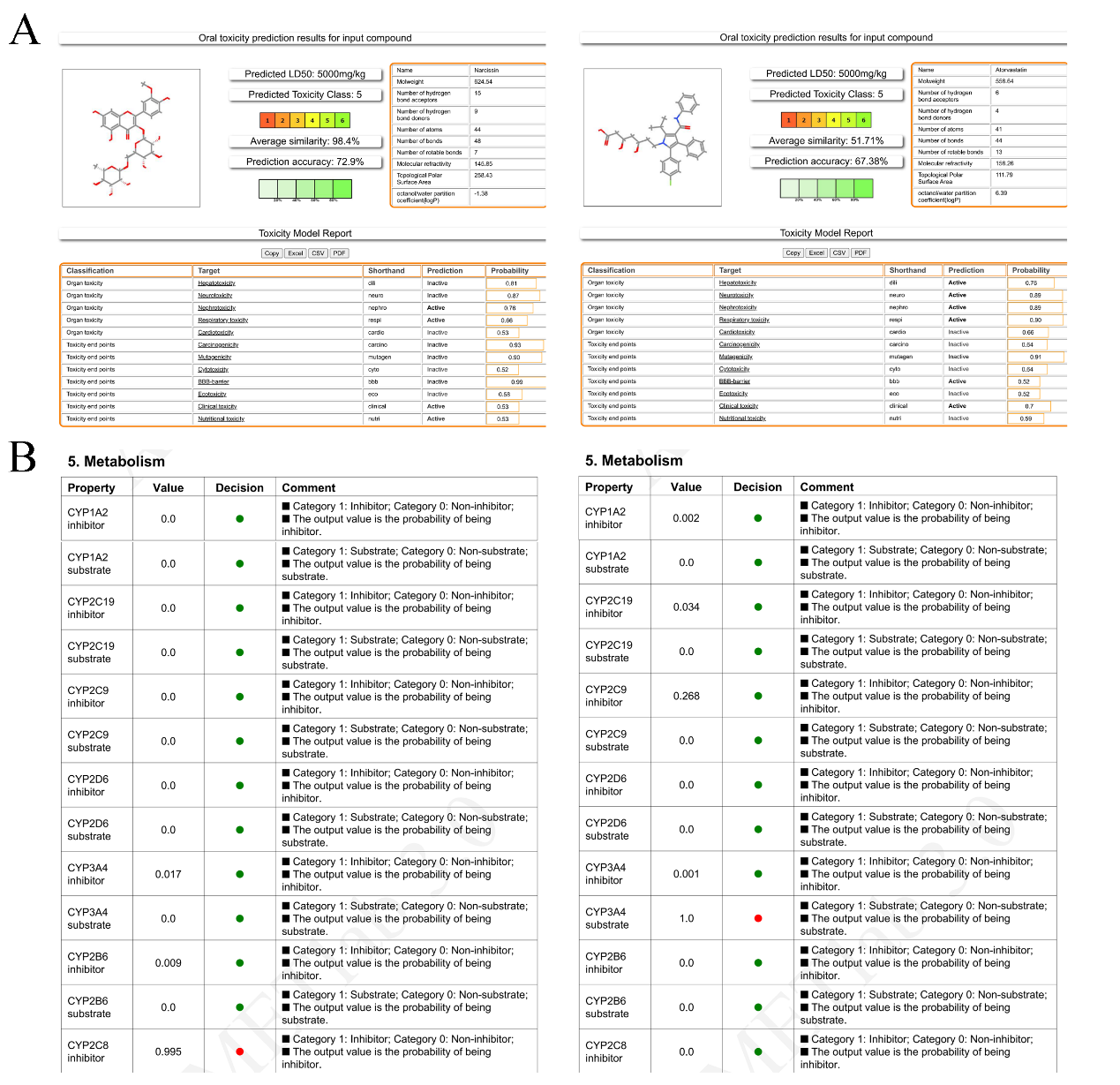


**Figure S1: Five Flavonoids isolated from G. pentaphyllum.** GF-1 (vicenin II), GF-2 (quercetin-3-*O*-*β*-D-neospheroside), GF-3 (Rutin), GF-4 (Narcissoside), GF-5 (kaempferide 3-*O*-neohesperidoside)

**Figure S2: Discovery of Narcissoside as a Potent Inhibitor of foam cell formation in GFs.** (A-B) THP-1 cells were stained with Oil Red O after exposing with ox-LDL (80 μg/mL) and treating by gypenosides (GP, 30 μg/mL), *G. pentaphyllum* flavonoids (GF, 30 μg/mL), GF-1 (vicenin II, 10 μM), GF-2 (quercetin-3-*O*-*β*-D-neospheroside, 10 μM), GF-3 (Rutin, 10 μM), GF-4 (Narcissoside, 10 μM), GF-5 (kaempferide 3-*O*-neohesperidoside, 10 μM) or Ato (atorvastatin, 1 μM) for 48 h, respectively (*n* = 3). (C) Comparison of THP-1 cells total cholesterol (TC) and triglycerides (TG) (*n* = 5). Data are expressed as mean ± SD (*n* ≥ 3). ^#^ *P* < 0.05, ^##^*P* < 0.01 *vs.* Con group; **P* < 0.05, ***P* < 0.01 *vs.* Mod group.

**Figure S3: NR4A1 is a potential transcription factor for CD36.** (A-B) Binding sites and binding sites of NR4A1.

**Figure S4: The toxicity and metabolism of Nar and Ato.** (A) The use of proTOX-3.0 to model the potential toxicity of Nar and Ato. (B) The use of ADMETlab 3.0 to model the potential metabolism of Nar and Ato.
